# Transactions of the Pathological Society of Philadelphia

**Published:** 1839-11-23

**Authors:** 


### MEDICAL EXAMINER.

DEVOTED TO MEDICINE, SURGERY, AND THE COLLATERAL SCIENCES.

No. 47.] PHILADELPHIA, SATURDAY, NOVEMBER 23, 1839. [Vol. II.

### TRANSACTIONS OF THE PATHOLOGICAL SOCIETY OF PHILADELPHIA.

*November 14th*, 1839.

The President, Dr. Gerhard, in the Chair.

Dr. Stillé presented a specimen of

*Strangulated Hernia*, and read the following account of the case:

S. S., æt. 58, a tailor, of temperate habits, has had a small elastic tumour of the right groin for about two years; it gave him no trouble till recently, and could be reduced at pleasure. He can assign no probable cause for its appearance other than the habitual constipation and the cross-legged posture common to persons of his trade. About the end of September last, he complained of a pain in the right groin, to his wife, who was then, for the first time, aware of the existence of any tumour in that part. The pain lasted but a few hours, and disappeared without any interference. S. then continued well until the morning of the 30th of October, when, being at his work, he suddenly felt a sharp and severe pain in the inguinal tumour. Supposing the pain to be colic, he went to stool, and had a slight passage, but without marked relief. He, however, continued to work with some difficulty until a little before his usual hour of leaving off, and hastened home. The pain now grew doubly severe, so much so that a physician was called in, who, according to the patient's subsequent declaration, pronounced that he had a rupture, and then ordered a dose of castor oil with a small white powder, having done which, he retired. This prescription procured no abatement of the symptoms, and no passage from the bowels took place; shortly after, vomiting came on, the matter rejected being principally stercoraceous. This condition continued during the whole night, while the patient was gradually becoming weaker, and his sufferings more violent. When morning came, the physician returned, and having found that an enema did not succeed in evacuating the bowels, directed the patient to be carried to the hospital, where he arrived about half past 9, A. M., on the 31st of October.

He lay, when I saw him, upon his right side, his knees drawn up, and his body curved towards them; he groaned deeply; his eyes were dull, his countenance haggard, his features contracted and livid; his extremities cold; his abdomen hard and moderately tumid. He was pulseless at the wrists, and referred his pain to the left hypochondrium and right groin. In the latter place, over the spermatic cord, there was a tumour about as large as an English walnut, elastic, moveable, and, in part, disappearing under pressure. The abdominal ring could be distinctly felt, and an irregular body lying over its lower margin. Some wine and water, with thirty drops of laudanum, were ordered, and a warm bath. After remaining in the bath for twenty minutes, his extremities felt as warm as the trunk; on leaving the water he fainted, and discharged from the rectum about a gill of yellow liquid fœces. On coming to himself, about two minutes afterwards, he was able to articulate faintly, but the pulsations of the radial artery were still imperceptible. Some more wine and water were administered, and he swallowed the mixture without difficulty. About 11 o'clock he was seen by Dr. Norris, then visiting surgeon of the hospital; his pulse might be felt pulsating faintly at the wrist. While preparations were making for performing the operation for strangulated hernia, the patient died.

On examining the body, about four hours after death, the following observations were made.

The tumour, in right groin, as before. The skin and superficial fascia were raised from the right iliac, inguinal and femoral regions, and the fibres of the cremaster muscles, found covering the tumour with a beautiful net work. The hernial sac was opened, and about one fluid drachm of reddish serum discharged from it. An intestinal loop was then disclosed, a little more than two inches long, of a dark and equable lake colour, a shining surface, and without any gangrenous points. The neck of the protruded fold of intestine was firmly embraced by the abdominal ring, and could not be withdrawn from it.* The cavity of the abdomen contained a considerable quantity of reddish serum, and the intestines were distended by fluid and gas. The former in the portion of small intestine, above the hernia, being of a grumous consistence, adherent to the mucous membrane, and convertible, by alcohol, into a dense fibrinous coagulum. The same is true of the matter contained in the loop without the abdomen.

This case adds one to the number already on record, going to prove the superior danger of small over large herniæ. It is perhaps probable that, had the person at first called to the case, been aware of this fact, he would have pursued a more energetic course of treatment, than the mere exhibition of palliatives. Had he done so, the fatal issue might, in all probability, have been averted.

* The epigastric artery passed on the inner side of the neck of the sac, and would not have been wounded by cutting upward from the stricture.

